# Supplementary material for: A spatio-temporally constrained gene regulatory network directed by PBX1/2 acquires limb patterning specificity via HAND2
Source: Nat Commun. 2023 Jul 6;14:3993. doi: 10.1038/s41467-023-39443-z (PMC10325989; doi:10.1038/s41467-023-39443-z)
Supplement: Supplementary file 1 — Supplementary Information [file 41467_2023_39443_MOESM1_ESM.pdf]

Supplementary Figures 1-9 and Supplementary Tables 1-3

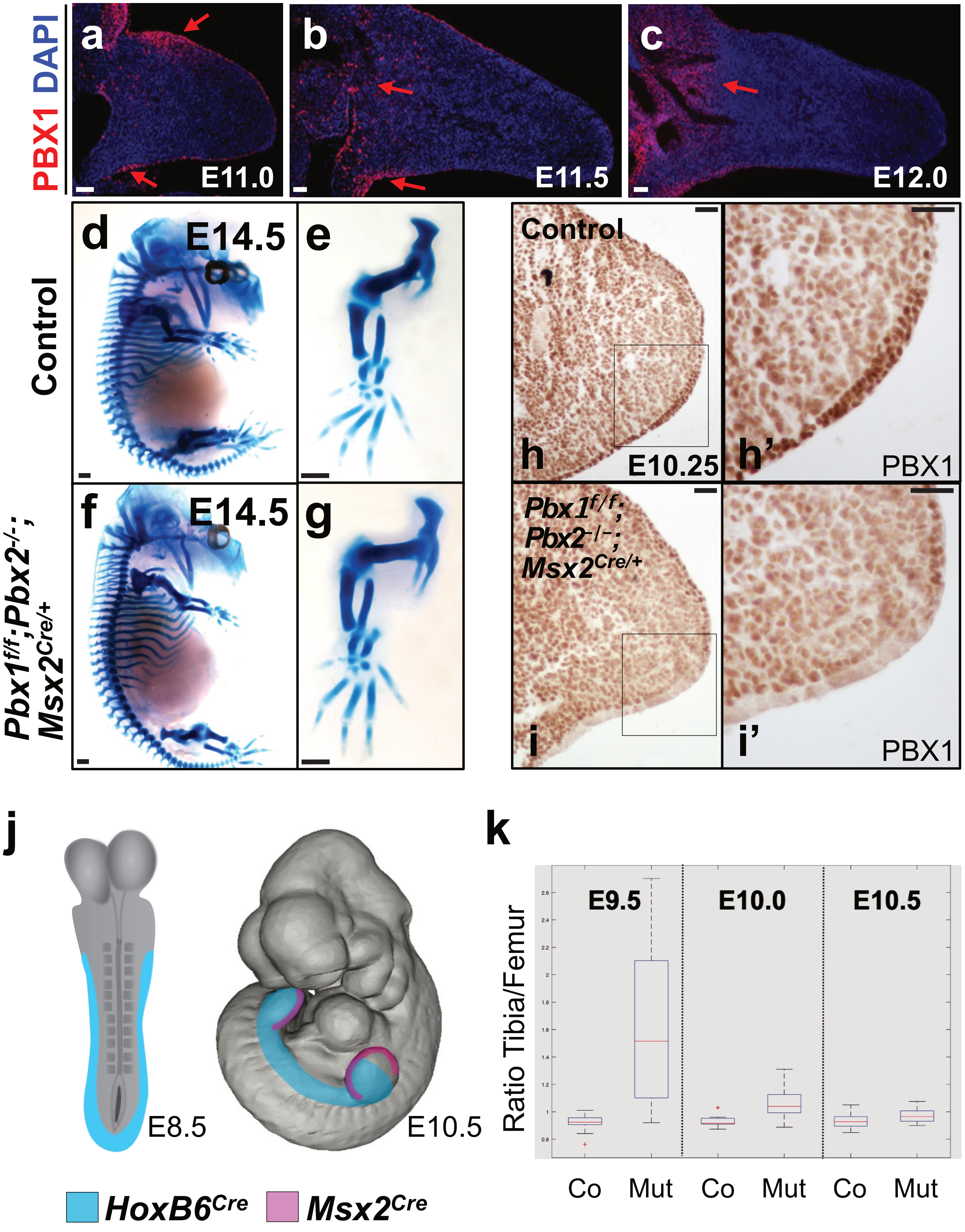

Supplementary Figure 1. Absence of limb skeletal abnormalities in compound mutant embryos with Pbx1 conditional loss in the apical ectodermal ridge on a Pbx2 null background. (a-c) Time-course showing PBX1 protein distribution (red immunofluorescence) in hindlimb buds (HLs) from E11.0 to E12.0. DAPI-counterstained nuclei (blue). n=3 samples were analyzed per developmental stage. Scale bars: 50 µm. (d-g) The skeleton cartilage of mutant and control littermate mouse embryos and hindlimb autopods (E14.5) is revealed by Alcian blue staining. *Pbx1*$^{f/f}$*;Pbx2*$^{-/-}$*;Msx2*$^{Cre/+}$: *Pbx1* was conditionally inactivated in the AER on a *Pbx2*-deficient background using a *Msx2Cre* deleter line. Numbers of samples analyzed per genotype reported in Supplementary Table 1. Scale bars: 500 µm. (h-h') Immunohistochemistry using a specific antibody detects PBX1 proteins in the AER and ectoderm in addition to its mesenchymal expression. Scale bars: 50 µm. (i,i') In contrast, PBX1 proteins are lost from the AER and ventral ectoderm in *Pbx1*$^{f/f}$*;Pbx2*$^{-/-}$*;Msx2*$^{Cre/+}$ HLs, consistent with activity of *Msx2Cre* (see j). n=3 samples were analyzed per genotype for h-i'. Scale bars: 50 µm. (j) Schematic representation of *Hoxb6Cre* and *Msx2Cre* activity, respectively, at E8.5 and E10.5. (k) Ratio of Tibia/Femur length in mutant *Pbx1*$^{f/f}$*;Pbx2*$^{-/-}$*;Hoxb6*$^{CreERT/+}$ embryos (Mut) compared to phenotypically wildtype control *Pbx1*$^{f/f}$*;Pbx2*$^{-/-}$ embryos (Co). Embryonic days (E) 9.5, E10.0 and E10.5 indicate the timepoints of tamoxifen injections. At least n=8 samples were analyzed per genotype and per developmental stage. Box plots indicate median, interquartile values, range and outliers.

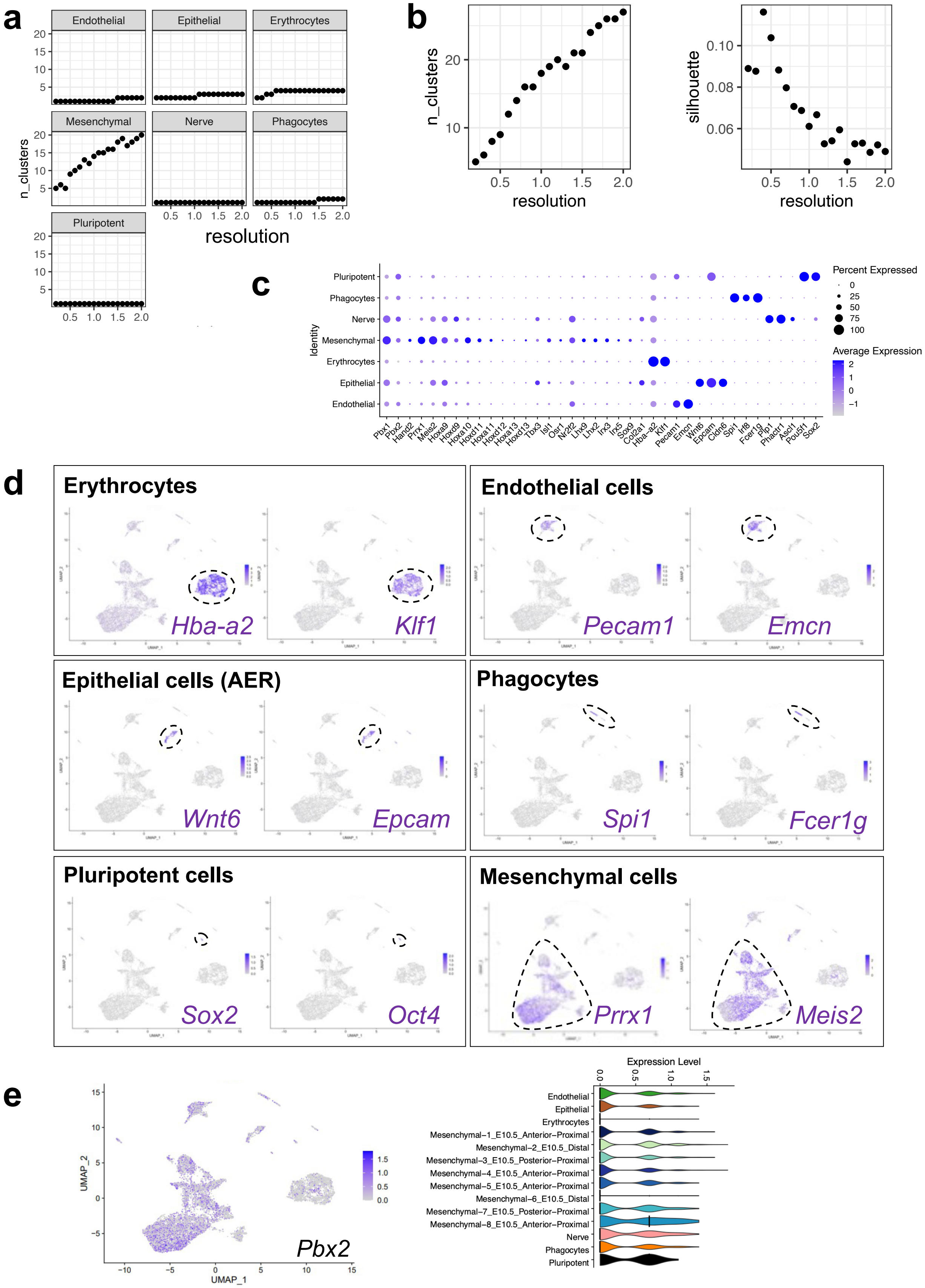

a
Endothelial
Epithelial
Erythrocytes
Mesenchymal
Nerve
Phagocytes
Pluripotent
n_clusters
resolution
b
n_clusters
resolution
silhouette
resolution
c
Percent Expressed
Average Expression
Identity
Pluripotent
Phagocytes
Nerve
Mesenchymal
Erythrocytes
Epithelial
Endothelial
Pbx1
Pbx2
Hand2
Prrx1
Meis2
Hoxa9
Hoxd9
Hoxa10
Hoxd10
Hoxa11
Hoxd11
Hoxd12
Hoxa13
Hoxd13
Tbx3
Isl1
Osr1
Nr2f2
Lhx9
Lhx2
Irx3
Irx5
Sox9
Col2a1
Hba-a2
Klf1
Pecam1
Emcn
Wnt6
Epcam
Cldn6
Spi1
Irf8
Fcer1g
Plp1
Phactr1
Ascl1
Pou5f1
Sox2
d
Erythrocytes
Hba-a2
Klf1
Endothelial cells
Pecam1
Emcn
Epithelial cells (AER)
Wnt6
Epcam
Phagocytes
Spi1
Fcer1g
Pluripotent cells
Sox2
Oct4
Mesenchymal cells
Prrx1
Meis2
e
UMAP_2
UMAP_1
Pbx2
Expression Level
Endothelial
Epithelial
Erythrocytes
Mesenchymal−1_E10.5_Anterior−Proximal
Mesenchymal−2_E10.5_Distal
Mesenchymal−3_E10.5_Posterior−Proximal
Mesenchymal−4_E10.5_Anterior−Proximal
Mesenchymal−5_E10.5_Anterior−Proximal
Mesenchymal−6_E10.5_Distal
Mesenchymal−7_E10.5_Posterior−Proximal
Mesenchymal−8_E10.5_Anterior−Proximal
Nerve
Phagocytes
Pluripotent

Supplementary Figure 2. Characterization of cell populations identified by scRNAseq of hindlimb buds at E10.5. (a) Scatter plots showing the number of clusters annotated to cell types of different lineages, at varying values of the resolution parameter. (b) Scatter plots showing the number of mesenchymal clusters (left panel) and the corresponding silhouette (metrics to assess cluster separation; right panel), at varying values of the resolution parameter. This analysis is focused only on mesenchymal cells. The maximum silhouette values correspond to a resolution of 0.4, resulting in eight mesenchymal cell clusters. (c) Dot plot of the most differentially expressed marker genes for each one of the seven main cell populations identified in the scRNAseq analysis of hindlimb buds. The size of each dot represents the proportion of cells within a given population that expresses the gene; the color intensity indicates the average expression level of the indicated gene. (d) UMAPs (color-coded based on gene expression levels) of the most differentially expressed marker genes identified for distinct cell populations: Erythrocytes (*Hba-a2, Klf1*); Endothelial cells (*Pecam1, Emcn*); Epithelial cells (*Wnt6, Epcam*); Phagocytes (*Spi1, Fcer1g*); and Pluripotent cells (*Sox2, Oct4*). (e) UMAP (color-coded based on gene expression levels; left) and violin plot (right) of normalized *Pbx2* expression.

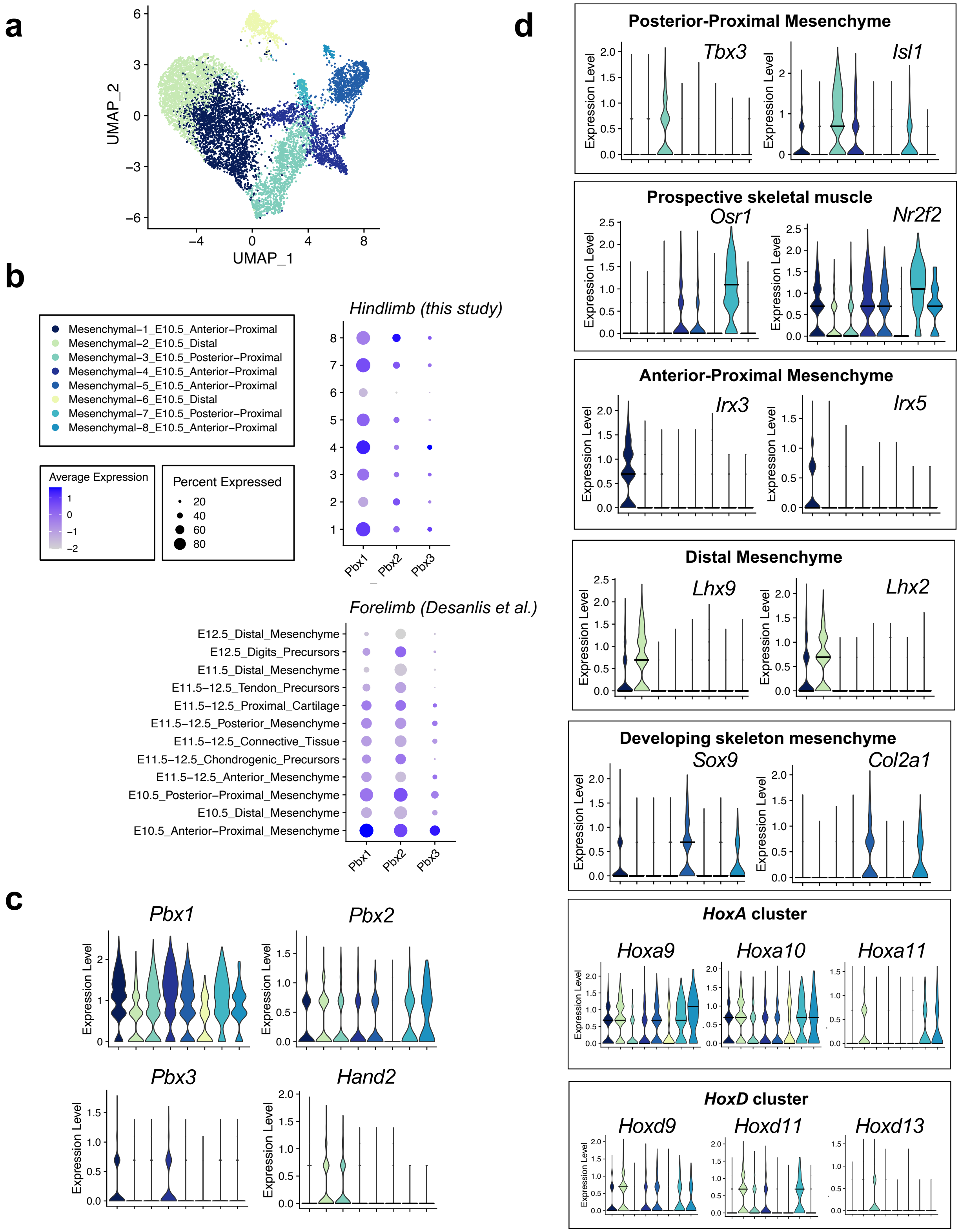
a
UMAP_1
UMAP_2
b
Mesenchymal−1_E10.5_Anterior−Proximal
Mesenchymal−2_E10.5_Distal
Mesenchymal−3_E10.5_Posterior−Proximal
Mesenchymal−4_E10.5_Anterior−Proximal
Mesenchymal−5_E10.5_Anterior−Proximal
Mesenchymal−6_E10.5_Distal
Mesenchymal−7_E10.5_Posterior−Proximal
Mesenchymal−8_E10.5_Anterior−Proximal
Average Expression
Percent Expressed
Hindlimb (this study)
Forelimb (Desanlis et al.)
E12.5_Distal_Mesenchyme
E12.5_Digits_Precursors
E11.5_Distal_Mesenchyme
E11.5−12.5_Tendon_Precursors
E11.5−12.5_Proximal_Cartilage
E11.5−12.5_Posterior_Mesenchyme
E11.5−12.5_Connective_Tissue
E11.5−12.5_Chondrogenic_Precursors
E11.5−12.5_Anterior_Mesenchyme
E10.5_Posterior−Proximal_Mesenchyme
E10.5_Distal_Mesenchyme
E10.5_Anterior−Proximal_Mesenchyme
Pbx1
Pbx2
Pbx3
c
Pbx1
Pbx2
Pbx3
Hand2
Expression Level
d
Posterior-Proximal Mesenchyme
Tbx3
Isl1
Prospective skeletal muscle
Osr1
Nr2f2
Anterior-Proximal Mesenchyme
Irx3
Irx5
Distal Mesenchyme
Lhx9
Lhx2
Developing skeleton mesenchyme
Sox9
Col2a1
HoxA cluster
Hoxa9
Hoxa10
Hoxa11
HoxD cluster
Hoxd9
Hoxd11
Hoxd13

Supplementary Figure 3. Characterization of the mesenchymal cell clusters identified by scRNAseq. (a) UMAP representation of the eight mesenchymal clusters in hindlimb buds that have been re-clustered without the other cell populations. (b) Dot plots show the average expression and the fraction of cells expressing the indicated genes (*Pbx1*, *Pbx2*, *Pbx3*) split by cluster of mesenchymal cells in hindlimb buds (this study) and forelimb buds (available scRNA-seq data from Desanlis et al. 2020, as described). (c) Violin plots showing the expression levels of key genes for this study (*Pbx1, Pbx2*, *Pbx3,* and *Hand2*) in hindlimb bud mesenchymal cell clusters at E10.5 (color-code identical to the UMAP in panel a). (d) Violin plots show the expression of mesenchyme-specific markers with known spatially restricted distributions and *HoxA* and *HoxD* genes in hindlimb bud mesenchymal cell clusters at E10.5 (color-code identical to the UMAP in panel a).

a

b

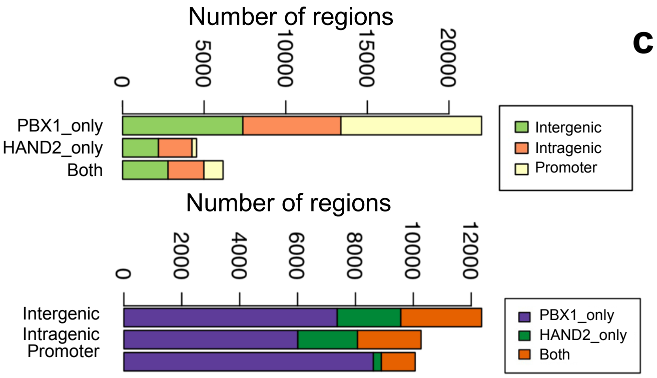

c

	Replicated peaks	Coverage (bp)
ATAC-seq	24,795	34,030,209
PBX1 ChIP-seq	28,177	43,497,077
HAND2 ChIP-seq	10,820	11,182,158
H3K27ac ChIP-seq	33,898	307,330,502
H3K27me3 ChIP-seq	9,866	89,606,534
CTCF ChIP-seq (*)	15,046	15,820,050

d e f g h

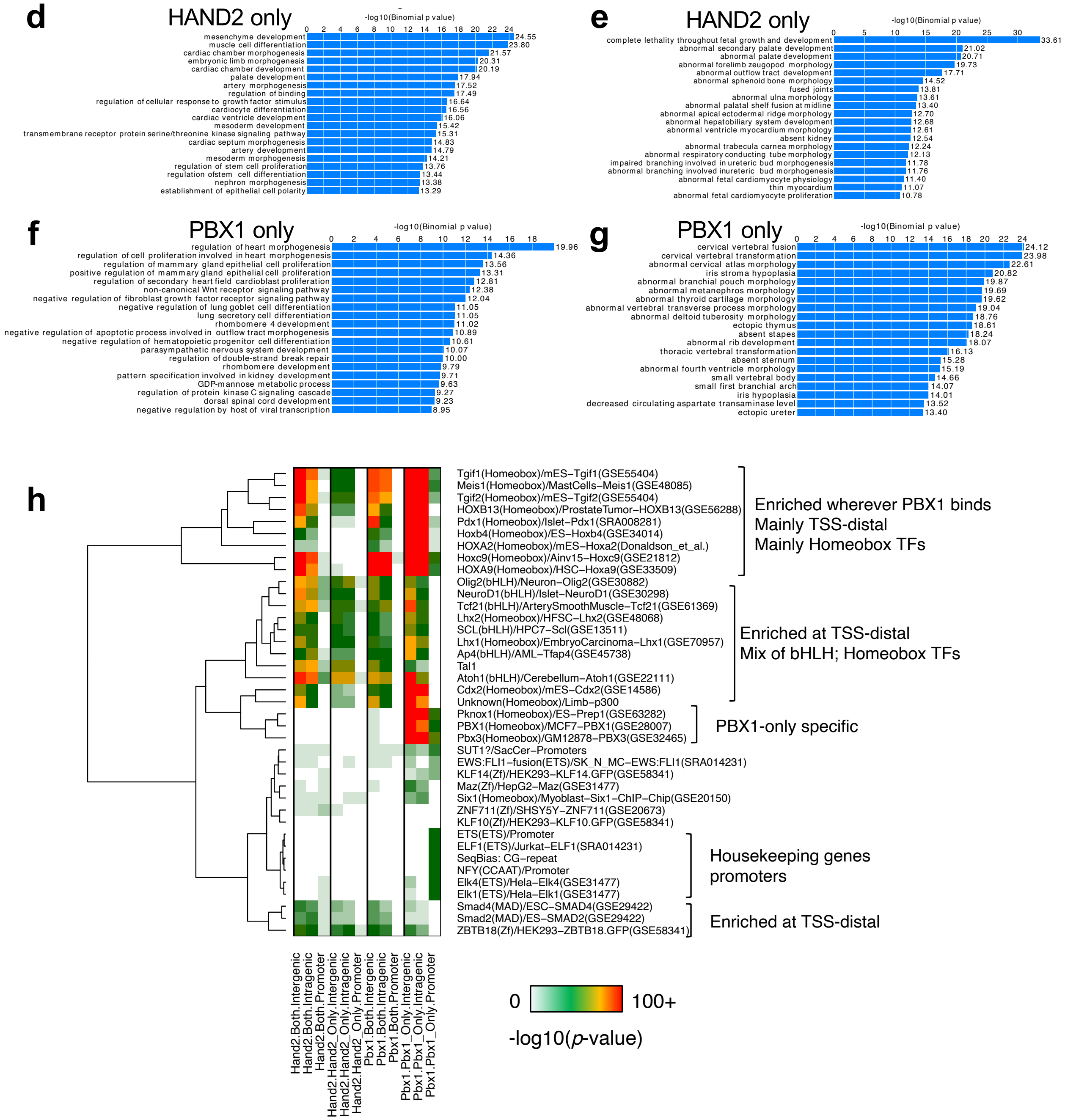

Supplementary Figure 4. Genome-wide analysis of PBX1, HAND2, and PBX1-HAND2 binding and chromatin landscapes in E10.5 mouse hindlimb buds. (a,b) Bar plots showing the distribution of peaks bound by PBX1-only, HAND2-only, or co-bound (Both) in relation to Intergenic, Promoter, or Intragenic genomic regions. (c) Table summarizing the number of replicated peaks and the coverage for all generated datasets. All datasets were generated in this study, except for the published CTCF ChIPseq. (d-g) Top enriched biological processes (d,f) and mouse phenotypes (e,g) associated with PBX1-only and HAND2-only peaks, respectively. Values on the x axis correspond to the uncorrected *p*-value of the enrichment. *P*-values from Binomial Tests. (h) Heatmap highlighting the enrichment of known transcription factor motifs in the regions bound by PBX1-HAND2 (Both), PBX1-only, or HAND2-only. Five subgroups are highlighted. The top bracket corresponds to transcription factor motifs correlated to PBX1 binding, mainly at TSS-distal domains that are associated to homeobox transcription factors. The second bracket corresponds to transcription factor motifs enriched at TSS-distal regions and comprising bHLH and homeobox transcription factors. The third bracket includes PBX1-specific cofactors, such as PREP1 and PBX3. The last bracket comprises SMAD transcription factor motifs, which are enriched at TSS-distal regions.

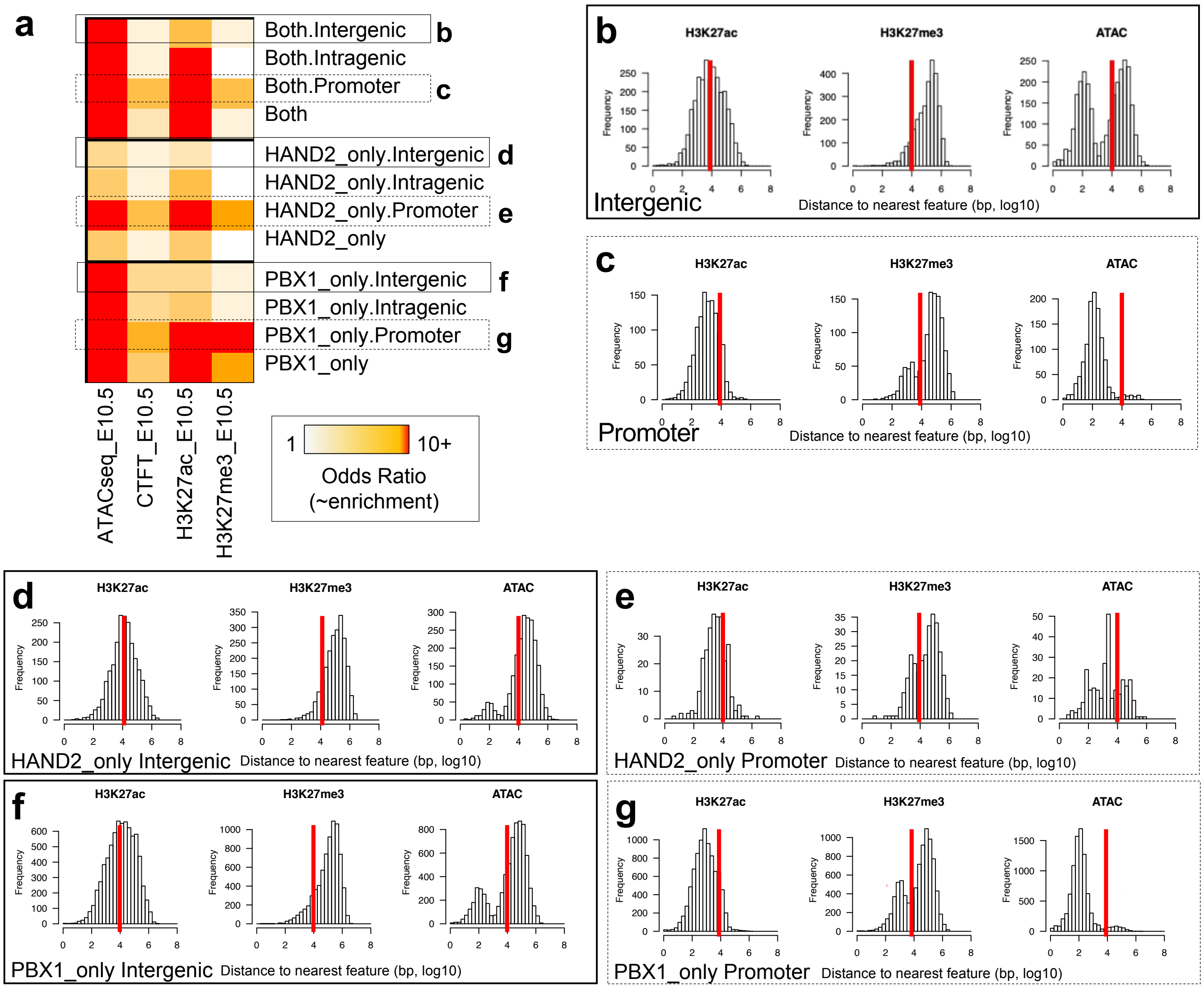

Supplementary Figure 5. Characterization of the chromatin state of regions bound by either PBX1 or HAND2 only, or by both factors. (a) Heatmap summarizing enrichment (as odds ratio of observed *versus* expected fraction of peaks) of the indicated chromatin features (columns) at peaks co-bound by both PBX1-HAND2 (Both), HAND2-only, or PBX1-only (rows). (b,c) Distributions of H3K27ac/H3K27me3 and ATACseq peaks relative to distance to nearest region bound by both PBX1-HAND2 at intergenic sites (panel b) or promoters (panel c). (d,e) Distribution of intergenic and promoter regions bound by HAND2 alone, relative to their distance to the nearest H3K27ac peak, H3K27me3-enriched region, or accessible region (measured by ATACseq). (f,g) Same analysis (as in panels d,e), including intergenic and promoter regions bound by PBX1 alone. A red vertical line in panels b-g is drawn at 10 kbp, to distinguish TSS-proximal from TSS-distal peaks.

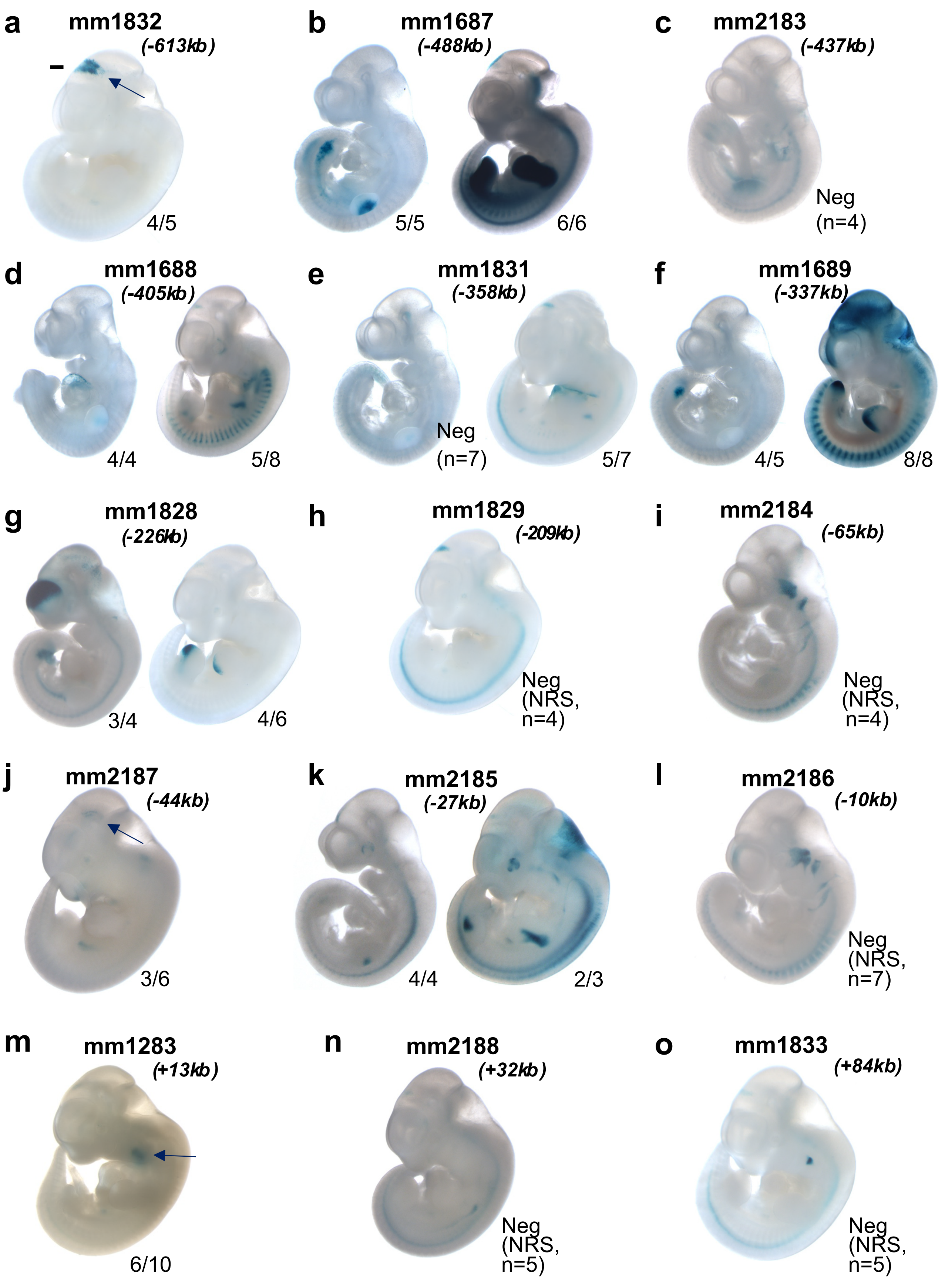

a
mm1832
(-613kb)
4/5
b
mm1687
(-488kb)
5/5
6/6
c
mm2183
(-437kb)
Neg
(n=4)
d
mm1688
(-405kb)
4/4
5/8
e
mm1831
(-358kb)
Neg
(n=7)
5/7
f
mm1689
(-337kb)
4/5
8/8
g
mm1828
(-226kb)
3/4
4/6
h
mm1829
(-209kb)
Neg
(NRS,
n=4)
i
mm2184
(-65kb)
Neg
(NRS,
n=4)
j
mm2187
(-44kb)
3/6
k
mm2185
(-27kb)
4/4
2/3
l
mm2186
(-10kb)
Neg
(NRS,
n=7)
m
mm1283
(+13kb)
6/10
n
mm2188
(+32kb)
Neg
(NRS,
n=5)
o
mm1833
(+84kb)
Neg
(NRS,
n=5)

Supplementary Figure 6. Comprehensive analysis of PBX1-bound *Hand2* limb enhancers by *LacZ* transgenic reporter assays. (a-o) Representative mouse embryos from *LacZ* transgenic reporter assays visualize in vivo enhancer activities at E10.5 and/or E11.5. X-gal staining reveals the spatial domains of enhancer activity in blue. Vista enhancer IDs (mm numbers) are listed on top of each panel and the relative distance of the enhancer to the *Hand2* TSS is indicated (in kb). Numbers at the bottom right of each embryo indicate the reproducibility of the spatial *LacZ* expression domain(s). Neg: negative. NRS: no reproducible staining. Scale bar (a-o): 100 μm; represented by black bar in top left corner of panel a**.**

a

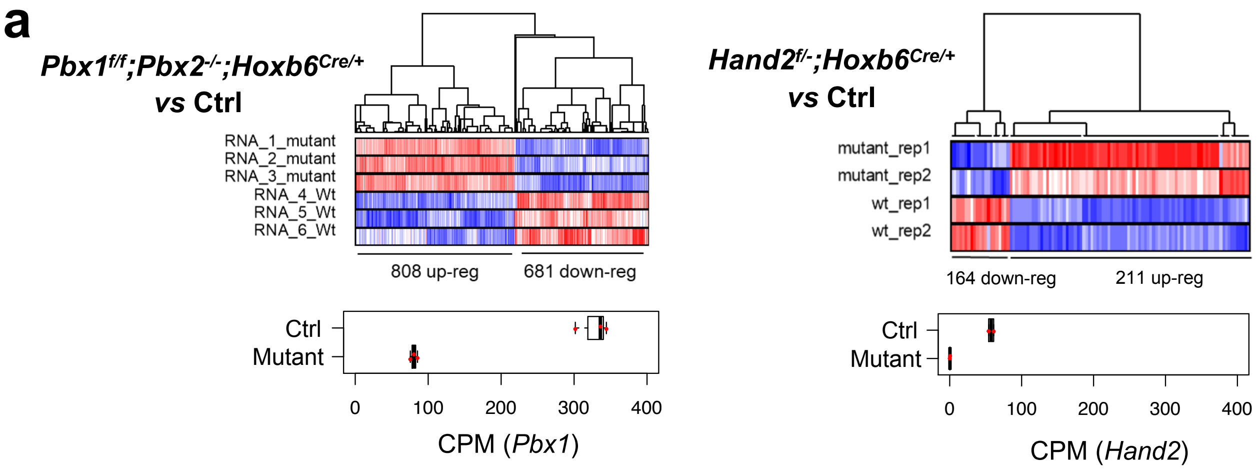

b

Pbx	**Up**	808	Not expressed in *Hand2* data	25
			Pbx only	719
			Both	46
			Discordant	18
	**Down**	681	Not expressed in *Hand2* data	17
			Pbx only	614
			Both	37
			Discordant	13
Hand	**Up**	211	Not expressed in *Hand2* data	15
			Hand2 only	137
			Both	46
			Discordant	13
	**Down**	164	Not expressed in *Hand2* data	16
			Hand2 only	93
			Both	37
			Discordant	18

c

Down-regulated	ECM; Cell Adhesion<br>Cytoskeleton	Both
	Digits morphogenesis<br>**Repressor TFs; Homeobox**	HAND2 Only
	ECM; Cell Adhesion<br>**TFs; Homeobox**<br>Neural Crest Cells Migration	PBX1 Only
Discordant	Hedgehog signaling pathway<br>Hindlimb morphogenesis<br>**TFs; Homeobox**	
Up-regulated	Development<br>**TFs**	Both
	Branch morphogenesis<br>Limb morphogenesis<br>Skeleton morphogenesis<br>**TFs; Homeobox**	HAND2 Only
	Development<br>Proximal-Distal pattern formation<br>**TFs; Homeobox**<br>Translation	PBX1 Only

d

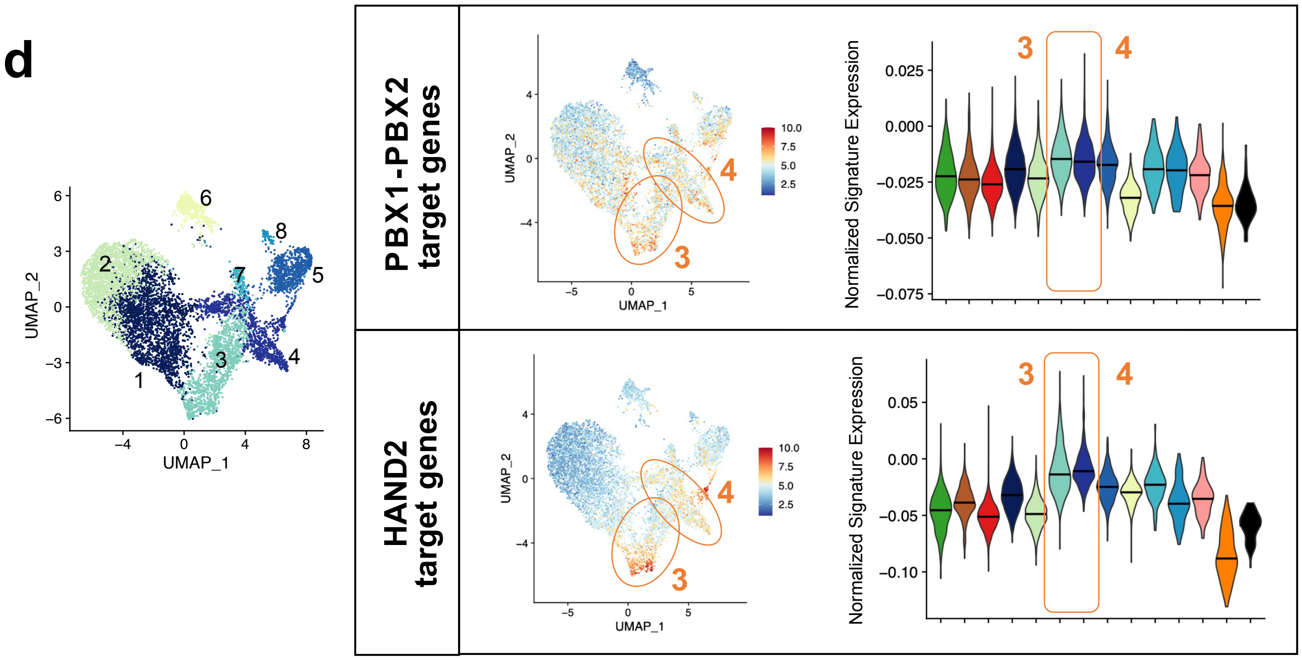

Supplementary Figure 7. Analysis of transcriptomes from whole *Pbx1/2* and *Hand2* mutant hindlimb buds. (a) Heatmap showing the relative expression (z-score) of DEGs identified by comparing the transcriptomes of wildtype (Ctrl) and *Pbx1/2* mutant (top left) or *Hand2* mutant (top right) mouse hindlimb buds (HLs) at E10.5. DEGs are genes whose expression is significantly changed between Ctrl and mutant samples (fold change equal or larger than 2, in either direction; FDR <= 0.05). Box plots show the counts per million (CPM) transcripts for *Pbx1* (bottom left; n = 3) and *Hand2* (bottom right; n = 2). Box plots indicate median, interquartile values, and range. Biological replicates for mutant and control samples in each box plot are indicated by red dots. (b) Table showing the numbers of upregulated (Up) and downregulated (Down) DEGs in *Pbx1/2* (*Pbx*) and *Hand2* deficient HLs, based only on the genes that were robustly detected in both sets of experiments. (c) Summary of the enriched GO terms associated with Biological Processes for genes that are up-regulated, down-regulated, or regulated in a discordant manner in HLs with different mutant genotypes. GO term categories are separated also based on binding by PBX1 only, HAND2 only or Both as determined by ChIPseq. (d) (Left) UMAP representation of 9,859 hindlimb cells assayed in scRNAseq of E10.5 HLs color-coded according to the enrichment of *Pbx1/2* and target genes using an available compendium (Dorothea). Analysis shows that *Pbx1/2* target genes exhibit highest enrichment in mesenchymal subclusters 3 and 4 (highlighted in orange; upper panel), which correspond to the two clusters showing also the highest enrichment of *Hand2* target genes (lower panel). (Right) Representation of the same data through violin plots (subclusters 3 and 4 highlighted in orange).

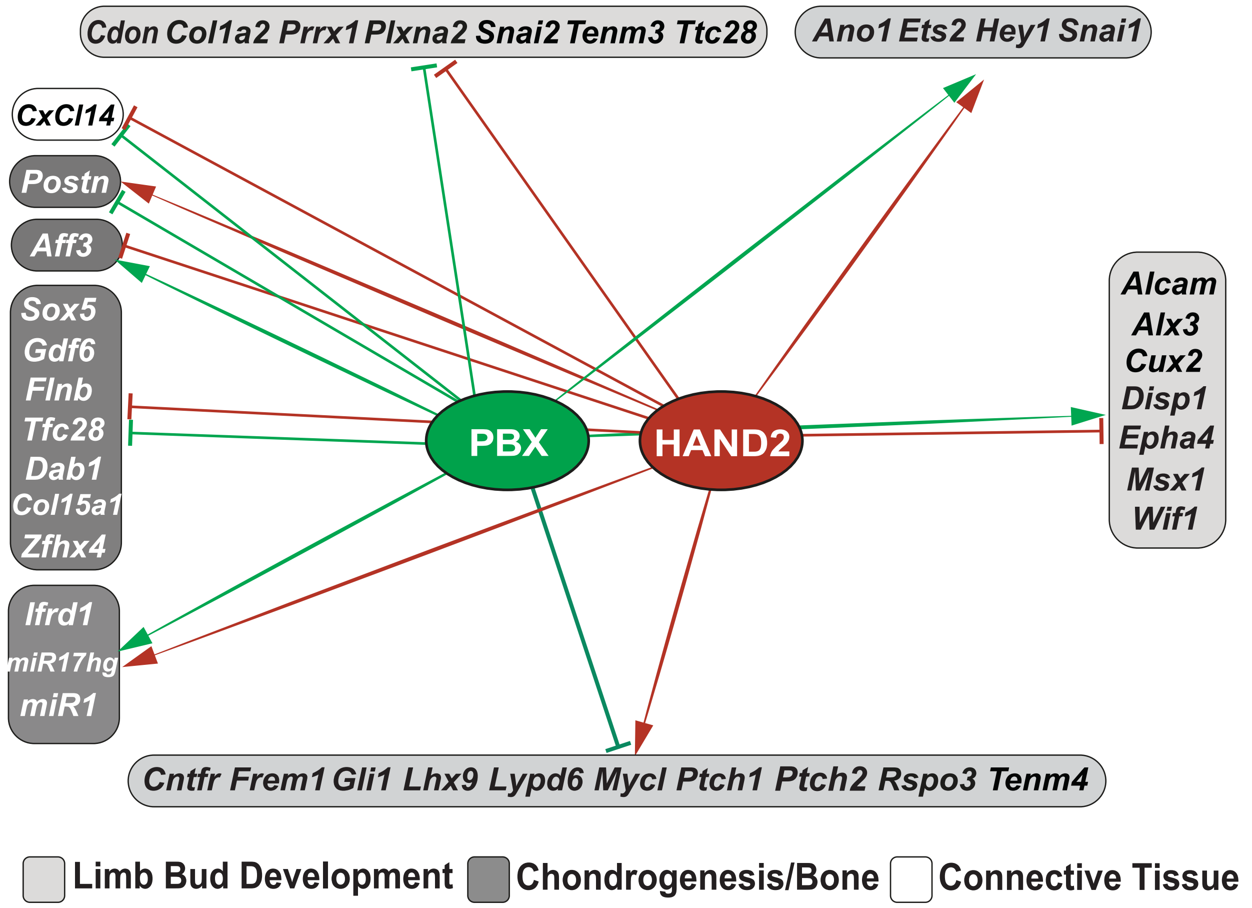

Supplementary Figure 8. The PBX-HAND2 controlled GRN operating in hindlimb buds. Construction of a PBX-HAND2 directed GRN from shared candidate target genes with known spatial expression in limb buds and/or essential functions during limb bud and skeletal development (see Supplementary Data 3). Shared candidate target genes were extracted from the bulk RNAseq datasets on hindlimb buds of *Pbx1cKOMes;Pbx2-/-* and *Hand2cKOMes* mutants compared to littermate controls. Concordant and discordant regulation of target genes by PBX and HAND2 are shown. PBX (green) and HAND2 (red) co-repress genes functioning in chondro-osteogenic differentiation (*Dab1*, *Col15a1*, *Flnb*,*Gdf6*, *Sox5*, *TcF28*, *Zfh4*) in parallel to positively regulating genes known to inhibit this differentiation (*Ifrd1*, *miR17hg*, *miR1*). Arrows: positive regulation; inhibitory lines (with bars): repression.

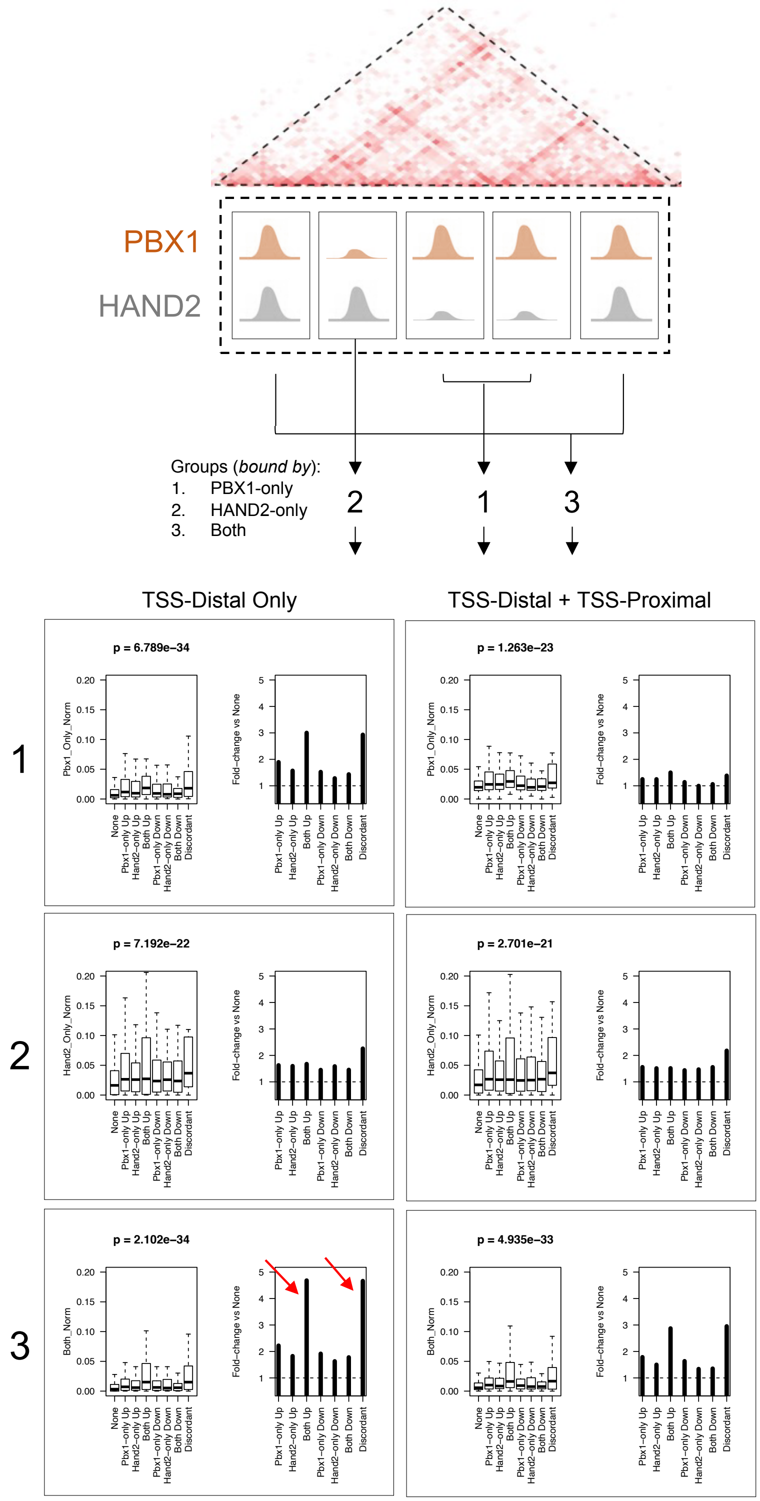
PBX1
HAND2
Groups (bound by):
1. PBX1-only
2. HAND2-only
3. Both
2
1
3
TSS-Distal Only
TSS-Distal + TSS-Proximal
1
p = 6.789e−34
p = 1.263e−23
2
p = 7.192e−22
p = 2.701e−21
3
p = 2.102e−34
p = 4.935e−33
Pbx1_Only_Norm
Hand2_Only_Norm
Both_Norm
Fold−change vs None
None
Pbx1−only Up
Hand2−only Up
Both Up
Pbx1−only Down
Hand2−only Down
Both Down
Discordant

Supplementary Figure 9. Integration of chromatin and transcriptomic datasets. TAD were scored based on the total number of regions bound by PBX1- or HAND2-only or by both PBX1 and HAND2 within each TAD, and the enrichment of the individual ChIPseq peaks. For each TAD, the scores were integrated separately for three groups (bound by PBX1- or HAND2-only, or both). For inter-TAD comparison, the scores were re-scaled based on the number of genes that are within the TAD and that are expressed in hindlimb buds. Each gene was assigned the TAD scores and the genes were separated based on RNAseq classification (X axis of the plots). A pair of plots is shown for each one of the three groups (rows), considering either TSS-distal only, or both TSS-distal and proximal peaks (columns). Box plots display the TAD scores for each group of genes, while the line plots indicate the fold-change of the scores in each group, compared to those of genes that show no differences in RNAseq datasets from mutant hindlimb buds. Red arrows highlight the group of genes whose TADs exhibit the highest scores, compared to control genes (“None”), which are harbored in TADs with one or more genes showing either concordant up-regulation, or discordant regulation, in the two types of mutants. Box plots indicate median, interquartile values, range, and outliers. *P*-values from Kruskal–Wallis one-way analysis of variance on the scores shown in the box plots.

a

Genotype	N	Gestational Days (E)	Skeletal Phenotype
$Pbx1^{f/+}$; $Pbx2^{+/-}$; $Msx2Cre^{+}$	15	10.5; 13.0; 14.5; P0	no phenotype
$Pbx1^{f/+}$; $Pbx2^{-/-}$; $Msx2Cre^{+}$	8	10.5; 13.0; 14.5; P0	no phenotype
$Pbx1^{f/f}$; $Pbx2^{+/-}$; $Msx2Cre^{+}$	12	10.5; 13.0; 14.5; P0	no phenotype
$Pbx1^{f/f}$; $Pbx2^{-/-}$; $Msx2Cre^{+}$	8	10.5; 13.0; 14.5; P0	no phenotype
$Pbx1^{f/-}$; $Pbx2^{+/-}$; $Msx2Cre^{+}$	6	13.5; P0	no phenotype
$Pbx1^{f/-}$; $Pbx2^{-/-}$; $Msx2Cre^{+}$	5	13.5; P0	no phenotype

b

Genotype	N	Gestational Days (E)	Skeletal Phenotype
Pbx1$^{f/+}$; *Pbx2*$^{+/-}$; *Hoxb6Cre*$^{+}$	10	10.5; 13.5; 15.5; 17.5	no phenotype
Pbx1$^{f/+}$; *Pbx2*$^{-/-}$; *Hoxb6Cre*$^{+}$	11	10.5; 13.5; 15.5	no phenotype
Pbx1$^{f/f}$; *Pbx2*$^{+/-}$; *Hoxb6Cre*$^{+}$	10	10.5; 13.5; 15.5	FL and HL defects
Pbx1$^{f/f}$; *Pbx2*$^{-/-}$; *Hoxb6Cre*$^{+}$	8	10.5; 13.5; 15.5	FL and HL defects

Supplementary Table 1. List of the total number of embryos analyzed with *Pbx1* conditional deletion in hindlimb buds using the *Msx2* and *Hoxb6* deleter lines, respectively, on a *Pbx2*-deficient background.

Hand2 enhancers: elements tested				
mm10 coordinates	Chr Start	Chr End	Distance to Hand2 TSS (in kb)	Vista ID
chr8	56706436	56707834	-613	mm1832
chr8	56831311	56833826	-488	mm1687
chr8	56883034	56884323	-437	mm2183
chr8	56914551	56916754	-405	mm1688
chr8	56961473	56963974	-358	mm1831
chr8	56983040	56984959	-336	mm1689
chr8	57093557	57095637	-226	mm1828
chr8	57110606	57112872	-209	mm1829
chr8	57254176	57256581	-65	mm2184
chr8	57275519	57277110	-44	mm2187
chr8	57292957	57294418	-27	mm2185
chr8	57327753	57329581	7	mm1284
chr8	57330807	57332981	10	mm2186
chr8	57333128	57334891	13	mm1283
chr8	57353269	57354301	32	mm2188
chr8	57359264	57363084	40	mm847
chr8	57404702	57405866	84	mm1833

Supplementary Table 2. List of *Hand2* enhancer elements tested in *LacZ* transgenic reporter assays. Coordinates, distance to the *Hand2* TSS, and Vista ID are indicated (mm10).

TOTAL PEAKS PER CHIP-SEQ	
PBX HL	18426
PBX BA2	31676
PBX MF	16851
HAND2 HL	8504
HOXA2 BA2	2319

INTERSECTION PBX HL vs MF vs BA2	Peaks	Percentage (%)
Unique PBX HL	6490	35.22
Unique PBX BA2	20045	63.28
Unique PBX MF	7651	45.40
Only PBX HL-BA2	4349	54.34
Only PBX HL-MF	1920	45.01
Only PBX MF-BA2	1615	43.20
Shared by all	5664	33.61
**INTERSECTION PBX HL vs PBX BA2 vs HAND2 HL**	**Peaks**	**Percentages**
Unique PBX HL	7056	38.29
Unique PBX BA2	20179	63.70
Unique HAND2 HL	2898	34.08
Only PBX HL-BA2	7305	54.35
Only PBX BA2-HAND2 HL	1443	48.84
Only PBX HL-HAND2 HL	1354	47.79
Shared by all	2710	31.87
**INTERSECTION PBX HL vs PBX BA2 vs HOXA2 BA2**	**Peaks**	**Percentages**
Unique PBX HL	8291	45.00
Unique PBX BA2	20921	66.05
Unique HOXA2 BA2	566	24.41
Only PBX HL-BA2	9136	54.35
Only PBX HL-HOXA2 BA2	121	43.08
Only PBX BA2-HOXA2 BA2	741	69.81
Shared by all	878	37.86
**INTERSECTION HOXA2 BA2 vs HAND2 HL**	**Peaks**	**Percentages**
Unique HOXA2 BA2	2030	87.54
Unique HAND2 HL	8215	96.60
Shared by all	289	12.46
**INTERSECTION PBX HL vs PBX MF vs HAND2 HL**	**Peaks**	**Percentages**
Unique PBX HL	8049	43.68
Unique PBX MF	8985	53.32
Unique HAND2 HL	4093	48.13
Only PBX HL-MF	6328	45.01
Only PBX HL-HAND2 HL	2797	47.66
Only PBX MF-HAND2 HL	282	18.09
Shared by all	1256	14.77

Supplementary Table 3. Numbers of replicated peaks for each ChIPseq across different embryonic tissues, indicating exclusive and shared peaks and their percentages. (Top panel) List of numbers of replicated peaks (“TOTAL PEAKS PER CHIP-SEQ”) for each duplicate ChIPseq dataset from hindlimb (HL), midface (MF) and branchial arch 2 (BA2). (Lower panels) Intersections between ChIPseq datasets as indicated. Peaks exclusive to each ChIP-seq are labelled as "Unique"; peaks overlapping only in two of the three datasets intersected as “Only”, e.g: "Only PBX HL-BA2"; peaks overlapping across the three datasets from the three different issues labelled as "Shared by all". For each intersection, the percentage of shared peaks is calculated for the smaller of the two ChIPseq datasets (as % of shared peaks relative to the total number of peaks).
