# Supplementary material for: A spatio-temporally constrained gene regulatory network directed by PBX1/2 acquires limb patterning specificity via HAND2
Source: Nat Commun. 2023 Jul 6;14:3993. doi: 10.1038/s41467-023-39443-z (PMC10325989; doi:10.1038/s41467-023-39443-z)
Supplement: Supplementary file 3 — Description of Additional Supplementary Files [file 41467_2023_39443_MOESM3_ESM.pdf]

Description of Additional Supplementary Data

File name: Supplementary Data 1
Description: **Complete lists of replicated ChIPseq peaks identified in wild-type mouse hindlimb buds for PBX1, HAND2, H3K27ac and H3K27me3, as well as of accessible chromatin regions defined by ATACseq.** Coordinates (chromosome, start and end) and combined p-value (as -log10) are shown. Combined p-values are obtained combining the MACS p-values of individual, biological replicates, using the Fisher's method.

File name: Supplementary Data 2
Description: **Lists of DEGs identified in *Pbx1cKO^{Mes};Pbx2$^{-/-}$* and *Hand2cKO^{Mes}* hindlimb buds in comparison to littermate controls plus intersection of RNAseq and ChIPseq datasets.** *Pbx_mutant* lists the results of the RNAseq data analysis performed with edgeR to compare *Pbx1cKO^{Mes};Pbx2$^{-/-}$* mutant to control hindlimb buds (HLs). The table shows, for each gene, the counts per million (CPM) for each replicate, along with the log2-fold-change, the *p*-value and the FDR. The last two columns indicate whether the gene was defined as up- or down-regulated in *Pbx* mutant HLs. *P*-values were calculated using the exact test as implemented in edgeR, which can be viewed as a generalization of the exact binomial test but generalized to over-dispersed counts. FDR were instead derived using the Benjamini-Hochberg correction for multiple tests. *Hand_mutant* lists the results of the RNAseq analysis on *Hand2cKO^{Mes}* mutant HLs. The table shows the data in the same order as for the *Pbx_mutant* dataset. *Summary* lists genes annotated as direct PBX1 and/or HAND2 targets based on the intersection of RNAseq with the ChIPseq datasets. For each gene, the table shows the log2-fold-change and FDR, separately for the *Pbx* and *Hand2* mutant hindlimb transcriptomes, along with the genes that were defined as significantly up- or down-regulated. *Regulation* lists the classification of each gene based on the behavior in the RNAseq profiles. The last six columns indicate whether the gene is a predicted direct PBX1 or HAND2 target, based on ChIPseq analysis. Target genes were identified by ChIPseq peaks being located either in proximity of the promoter (within 2.5 kb) or within 10 to 100 kb from the main annotated TSS. *Summary_Numbers* illustrates the breakdown of the DEG counts in the mutant limbs, based on how they have been classified (see *Summary*). Summary_100kbp and Summary_10kbp show the list of the total number of ChIPseq peaks near DEGs identified by RNAseq within a 100kb and 10kb window, respectively.

File name: Supplementary Data 3
Description: **Functional annotation of candidate target genes shared between PBX and HAND2.** All candidate target genes were extracted from the bulk RNAseq datasets on hindlimb buds of *Pbx1cKO^{Mes};Pbx2$^{-/-}$* and *Hand2cKO^{Mes}* mutants compared to littermate controls. The information on expression of candidate target genes in limb bud and associated limb phenotypes was obtained and manually curated from the MGI (https://www.informatics.jax.org) and EMBRYS databases (https://www.embrys.jp), except where indicated otherwise by doi.org links in the Table. Blue colors indicate genes with known limb bud spatial expression patterns, orange colors indicate genes with associated limb phenotypes. These two types of annotated genes were used to generate the comprehensive PBX-HAND2 GRN shown in Supplementary Fig. 8.
