# Supplementary material for: A spatio-temporally constrained gene regulatory network directed by PBX1/2 acquires limb patterning specificity via HAND2
Source: Nat Commun. 2023 Jul 6;14:3993. doi: 10.1038/s41467-023-39443-z (PMC10325989; doi:10.1038/s41467-023-39443-z)
Supplement: Supplementary file 7 — Reporting Summary [file 41467_2023_39443_MOESM7_ESM.pdf]

nature portfolio

Corresponding author(s): Licia Selleri

Last updated by author(s): May 15, 2023

Reporting Summary

Nature Portfolio wishes to improve the reproducibility of the work that we publish. This form provides structure for consistency and transparency in reporting. For further information on Nature Portfolio policies, see our Editorial Policies and the Editorial Policy Checklist.

Statistics

For all statistical analyses, confirm that the following items are present in the figure legend, table legend, main text, or Methods section.

n/a	Confirmed	
☐	☒	The exact sample size (*n*) for each experimental group/condition, given as a discrete number and unit of measurement
☐	☒	A statement on whether measurements were taken from distinct samples or whether the same sample was measured repeatedly
☐	☒	The statistical test(s) used AND whether they are one- or two-sided<br>*Only common tests should be described solely by name; describe more complex techniques in the Methods section.*
☐	☒	A description of all covariates tested
☐	☒	A description of any assumptions or corrections, such as tests of normality and adjustment for multiple comparisons
☐	☒	A full description of the statistical parameters including central tendency (e.g. means) or other basic estimates (e.g. regression coefficient) AND variation (e.g. standard deviation) or associated estimates of uncertainty (e.g. confidence intervals)
☐	☒	For null hypothesis testing, the test statistic (e.g. *F*, *t*, *r*) with confidence intervals, effect sizes, degrees of freedom and *P* value noted<br>*Give P values as exact values whenever suitable.*
☒	☐	For Bayesian analysis, information on the choice of priors and Markov chain Monte Carlo settings
☒	☐	For hierarchical and complex designs, identification of the appropriate level for tests and full reporting of outcomes
☐	☒	Estimates of effect sizes (e.g. Cohen's *d*, Pearson's *r*), indicating how they were calculated

Our web collection on statistics for biologists contains articles on many of the points above.

Software and code

Policy information about availability of computer code

Data collection	No specific software was used for data collection.
Data analysis	R v3; cellRanger v2.2.0; R packages (tidyverse v1.3; Seurat v4.1; clusterProfiler v3.18; msigdbr v7.2.1; dorothea v1.2.1; circlize v0.4.12; ComplexHeatmap v2.6.2; gtools v3.8.2); fastqc v0.11.9; bowtie2 v1.2; tophat v2.0.13; samtools v0.1.18; MACS v1.413; MPSC (https://genometric.github.io/MSPC/); wigToBigWig; HOMER (v4.9); htseq-count; leiden; custom R and python scripts

For manuscripts utilizing custom algorithms or software that are central to the research but not yet described in published literature, software must be made available to editors and reviewers. We strongly encourage code deposition in a community repository (e.g. GitHub). See the Nature Portfolio guidelines for submitting code & software for further information.

Data

Policy information about availability of data

All manuscripts must include a data availability statement. This statement should provide the following information, where applicable:

- Accession codes, unique identifiers, or web links for publicly available datasets
- A description of any restrictions on data availability
- For clinical datasets or third party data, please ensure that the statement adheres to our policy

ChIPseq, ATACseq, scRNAseq and RNAseq datasets have been deposited in the NCBI GEO database under the identifier GSE197859 (https://www.ncbi.nlm.nih.gov/

geo/query/acc.cgi?acc=GSE197859). Pre-processed scRNAseq data have also been deposited in Zenodo (https://zenodo.org/record/7884496#.ZFA4dexBzvU). Additional data supporting the reported findings are available from the corresponding author upon request.

Human research participants

Policy information about studies involving human research participants and Sex and Gender in Research.

Reporting on sex and gender	N/A
Population characteristics	N/A
Recruitment	N/A
Ethics oversight	N/A

Note that full information on the approval of the study protocol must also be provided in the manuscript.

Field-specific reporting

Please select the one below that is the best fit for your research. If you are not sure, read the appropriate sections before making your selection.

☒ Life sciences ☐ Behavioural & social sciences ☐ Ecological, evolutionary & environmental sciences

For a reference copy of the document with all sections, see nature.com/documents/nr-reporting-summary-flat.pdf

Life sciences study design

All studies must disclose on these points even when the disclosure is negative.

Sample size

Sample size are based on standards in the field. All omics-datasets were collected following ENCODE guidelines stating that "experiments should be performed with two or more biological replicates". For ATACseq and bulk RNAseq, 3 biological replicates were analyzed given that it required less numbers of embryos. ChIPseq was performed used pools of 60-80 embryonic hindlimb buds per replicate. The high quality and reproducibility of both replicates resulted statistical significance of the peaks called. In addition it is standard to analyze two ChIPseq. See the the ENCODE guidelines: https://www.encodeproject.org/about/experiment-guidelines/.
To minimize batch-to-batch variation for scRNAseq, the dataset at E10.5 was collected using 10 wild-type pooled embryos.

For whole mount RNA situ hybridization, RNAScope, immunofluorescense and LacZ reporter assays in transgenic founder embryos minimally 3 independent biological replicates were analyzed per genotype and developmental stage.

Data exclusions

No data were excluded

Replication

Only data that could be replicated in at least 2 biological replicates were included in the study.

All omics-datasets were collected following ENCODE guidelines, which indicates that "experiments should be performed with two or more biological replicates". For ATACseq and bulk RNAseq, three biological replicates were conducted given that it required less numbers of embryos. ChIPseq was performed pooling 60-80 embryonic hindlimb buds per replicate. Given the high quality and reproducibility of both replicates, it was enough to analyze two replicates to achieve statistical significance of the number of peaks called using those 2 replicates. Two ChIPseq replicates are the standard in the field (see e.g. the ENCODE guidelines https://www.encodeproject.org/about/experiment-guidelines/).
scRNAseq datasets were obtained from a single pool of 10 embryos at the same gestational day.

For whole mount RNA situ hybridization, RNAScope, immunofluorescense and LacZ reporter assays in transgenic founder embryos minimally 3 independent biological replicates were analyzed per genotype and developmental stage. The number of founder embryos for each LacZ transgenic reporter assay are shown in Figure 4. Whole mount RNA in situ hybridization and skeletal preparations: n>3 samples per genotype and stage isolated from different females were analyzed in minimally two completely independent experiments.

Randomization

Randomization is not possible, as due to the genetic complexity of the analysis mouse embryos have to be genotyped prior to analysis. Given the complexity of some of the genetic interactions, it was not possible to maintain a unique genetic background for these studies.

Blinding

For lacZ reporter activities embryos were stained and expression patterns scored prior to determining the genotypes (transgenic versus non-transgenic embryos).
For genome-wide analysis, such as bulk or scRNAseq, ChIP-seq and ATAC-seq blinding is not required (e.g. ENCODE guidelines https://www.encodeproject.org/about/experiment-guidelines/).

Reporting for specific materials, systems and methods

We require information from authors about some types of materials, experimental systems and methods used in many studies. Here, indicate whether each material, system or method listed is relevant to your study. If you are not sure if a list item applies to your research, read the appropriate section before selecting a response.

Materials & experimental systems

n/a	Involved in the study
☐	☒ Antibodies
☒	☐ Eukaryotic cell lines
☒	☐ Palaeontology and archaeology
☐	☒ Animals and other organisms
☒	☐ Clinical data
☒	☐ Dual use research of concern

Methods

n/a	Involved in the study
☐	☒ ChIP-seq
☒	☐ Flow cytometry
☒	☐ MRI-based neuroimaging

Antibodies

Antibodies used	Antibodies used for ChIPseq were: PBX1 (Cell Signaling, #4243S), FLAG for HAND2 (Sigma, F1804), H3K27ac (Abcam, ab4729) and H3K27me3 (Millipore, #07-449). In all cases, a total of 5ug of antibodies were used in each ChIPseq experiment.<br>Primary antibodies used for immunofluorescence were: PBX1 (Cell Signalling, #4342, dilution used 1:200) and FLAG M2 (Sigma, F1804, dilution used 1:500). Primary antibody binding was detected by AlexaFluor-conjugated secondary antibodies (Invitrogen, dilution used 1:400-1:1000).<br>For Co-immunoprecipitation experiments, 5ug of antibodies were used for PBX1 (Cell Signaling, #4243S), FLAG for HAND2 (Sigma, F1804) and normal rabbit IgG (R&D AB-105-C) for controls per sample. Western blot analyses were conducted with antibodies against HAND2 (Santa Cruz Biotechnology A-12, dilution 1:1000), PBX1 (Cell Signaling #4243S, dilution 1:5000) and FLAG (Sigma F1804, dilution 1:2000).
Validation	PBX and HAND2 antibodies were initially validated on mutant mouse tissues lacking the respective transcription factors.<br>The specificity of all other commercial antibodies were validates by their manufacturers (for details see the respective websites).<br>1. The PBX1 Ab was validated using mouse mid-face tissues and by ChIPqPCR experiments (Losa et al., 2018).<br>2. The anti-histone H3 (acetyl K27) Ab was validated by the manufacturer in HELA cells by ChIPqPCR.<br>3. The monoclonal anti-FLAG M2 antibodies were validated using a wild-type limb buds for ChIPseq analysis to detect non-specifically enriched peaks (Osterwalder et al. 2014 10.1016/j.devcel.2014.09.018).<br>4. The anti-histone H3 (trimethylated K27) antibody has been used by many groups for ChIPseq analysis (see e.g. Yukawa et al, 2014; Barrero et al., 2013).<br>5. The antibodies used for Western Blot have been validated in extensively: anti-FLAG (see e.g. Osterwalder et al. 2014 10.1016/j.devcel.2014.09.018) anti-Hand2 antibodies (see e.g. Prummel et al., 2022; Zhang et al., 2023) and the PBX1 antibodies (see e.g Welsh et al., 2018; Losa et al., 2018).

Animals and other research organisms

Policy information about studies involving animals; ARRIVE guidelines recommended for reporting animal research, and Sex and Gender in Research

Laboratory animals	All loss-of-function mice for Pbx genes and Hand2 were bred in mixed genetic backgrounds. Wild-type (Swiss Webster) mice of about 8 weeks were purchased from Charles River Laboratories and time-mated to obtain wild-type embryos at gestational day E10.5 (noon of the day of the plug was E0.5). Mouse embryos were collected from pregnant females after euthanasia and confirmed death.
Wild animals	No wild animals were used.
Reporting on sex	All mouse embryos irrespective of their sex were used for analysis.
Field-collected samples	No field-collected samples were used
Ethics oversight	Experiments using Pbx1/Pbx2 mice and embryos were performed following the Weill Cornell Medical College and UCSF IACUC guidelines for housing, husbandry, and welfare. Animal studies conducted in Switzerland involving Hand2 and Pbx/Hand2 compound mutant mice and embryos were approved by the Regional Commission on Animal Experimentation and the Basel Cantonal Veterinary Office. Mouse transgenesis work at Lawrence Berkeley National Laboratory (LBNL) was approved by the LBNL Animal Welfare Committee.

Note that full information on the approval of the study protocol must also be provided in the manuscript.

ChIP-seq

Data deposition

☒ Confirm that both raw and final processed data have been deposited in a public database such as GEO.

☒ Confirm that you have deposited or provided access to graph files (e.g. BED files) for the called peaks.

Data access links
May remain private before publication.

GSE197859 (https://www.ncbi.nlm.nih.gov/geo/query/acc.cgi?acc=GSE197859; Token: edqfkysonzitpif)

Files in database submission

For each one of the sample listed below (including input DNAs), unfiltered, untrimmed fastq files were submitted to GEO along with unreplicated peaks called using MACS v1.4 (in bed format).

GSM5931400 H3K27ac ChIP-seq Replicate 1
GSM5931401 H3K27ac ChIP-seq Replicate 2
GSM5931402 H3K27me3 ChIP-seq Replicate 1
GSM5931403 H3K27me3 ChIP-seq Replicate 2
GSM5931404 Hand2 ChIP-seq Replicate 1
GSM5931405 Hand2 ChIP-seq Replicate 2
GSM5931406 Pbx1 ChIP-seq Replicate 1
GSM5931407 Pbx1 ChIP-seq Replicate 2
GSM5931408 H3K27ac ChIP-seq Input DNA Replicate 1
GSM5931409 H3K27ac ChIP-seq Input DNA Replicate 2
GSM5931410 H3K27me3 ChIP-seq Input DNA Replicate 1
GSM5931411 H3K27me3 ChIP-seq Input DNA Replicate 2
GSM5931412 Hand2 ChIP-seq Input DNA Replicate 1
GSM5931413 Hand2 ChIP-seq Input DNA Replicate 2
GSM5931414 Pbx1 ChIP-seq Input DNA Replicate 1
GSM5931415 Pbx1 ChIP-seq Input DNA Replicate 2

Genome browser session
(e.g. UCSC)

N/A

Methodology

Replicates

2 biological replicates each

Sequencing depth

Refer to GSE197859

Antibodies

Refer to GSE197859

Peak calling parameters

macs14 -t [..] -c [..] --name=[..] --format=SAM --gsize=mm --bw=150 --nomodel --shiftsize=100 -w -S

Data quality

Refer to bed files in GSE197859

Software

ChIPseq reads were aligned to the mm10 release of the mouse genome (Dec. 2011, GRCm38) using
Bowtie (v1.2) with parameters -v 2 -m 1. Peak calling was performed using Model-Based Analysis for ChIPseq (MACS) v1.413 with matched input DNA as control and parameters --gsize=mm --bw=150 --nomodel --shiftsize=100. Each experiment was performed in duplicate; for HAND2 and PBX1 ChIPseq analysis, peaks detected in both replicates were merged using a statistical method that takes into account the combined statistical evidence from the two replicates14 (MSPC https://genometric.github.io/MSPC/; parameters: -r biological -s 1E-10 -W 1E-6).
